# Childhood- versus Adolescent-Onset Antisocial Youth with Conduct Disorder: Psychiatric Illness, Neuropsychological and Psychosocial Function

**DOI:** 10.1371/journal.pone.0121627

**Published:** 2015-04-02

**Authors:** Vicki A. Johnson, Andrew H. Kemp, Robert Heard, Christopher J. Lennings, Ian B. Hickie

**Affiliations:** 1 Clinical Research Unit, Brain & Mind Research Institute, University of Sydney, 100 Mallet Street, Camperdown, 2050, Sydney, Australia; 2 University Hospital and Faculty of Medicine, University of São Paulo, São Paulo, Brazil; 3 School of Psychology & Discipline of Psychiatry, University of Sydney, Sydney, Australia; 4 Lennings Seidler Collins Psychology: Clinical and Forensic Psychology Services, Level 5, 154 Elizabeth St., Sydney, Australia and Faculty of Policing and Law Enforcement, Charles Sturt University, Sydney, Australia; Merced, UNITED STATES

## Abstract

**Objective:**

The present study investigates whether youths with childhood-onset antisocial behavior have higher rates of psychiatric illness, neuropsychological and psychosocial dysfunction than youths who engage in antisocial behavior for the first time in adolescence. Prior studies have generally focused on single domains of function in heterogeneous samples. The present study also examined the extent to which adolescent-onset antisocial behavior can be considered normative, an assumption of Moffitt’s dual taxonomy model.

**Method:**

Forty-three subjects (34 males, 9 females, mean age = 15.31, age range 12–21) with a diagnosis of conduct disorder (CD) were recruited through Headspace Services and the Juvenile Justice Community Centre. We compared childhood-onset antisocial youths (n = 23) with adolescent-onset antisocial youths (n = 20) with a conduct disorder, across a battery of psychiatric, neuropsychological and psychosocial measures. Neuropsychological function of both groups was also compared with normative scores from control samples.

**Results:**

The childhood-onset group displayed deficits in verbal learning and memory, higher rates of psychosis, childhood maltreatment and more serious violent behavior, all effects associated with a large effect size. Both groups had impaired executive function, falling within the extremely low range (severely impaired).

**Conclusions:**

Childhood-onset CD displayed greater cognitive impairment, more psychiatric symptoms and committed more serious violent offences. The finding of severe executive impairment in both childhood- and adolescent-onset groupings challenges the assumption that adolescent-onset antisocial behavior is a normative process.

## Introduction

Findings in the youth offender literature indicate that psychiatric symptoms, neuropsychological deficits and psychosocial factors play a role in the aetiology and maintenance of violence and aggression [1–7]. However, the extant literature is characterized by a variety of limitations [8] including a focus on adult incarcerated populations, defining age of onset by first criminal charge (rather than initial behavioral difficulties), disorder heterogeneity and a focus on discrete and singular domains of function (rather than a profile of deficits). Here we examine differences between adolescents with either childhood- or adolescent-onset conduct disorder across a battery of psychiatric, neuropsychological and psychosocial measures.

### The Prevalence of Mental Illness in Violent Offenders

Mental health problems are over-represented amongst incarcerated adults and youths in several countries, with rates higher than in the general community [9–13]. High rates of psychiatric comorbidity have been reported in child and adolescent community samples [14–17], and the prevalence of risk factors such as substance use [18–21] and a history of violence [22–23] are also high.

### Neuropsychological Deficits in Delinquent Youth

Areas of cognitive function most frequently identified as showing deficits in delinquent youth include IQ, verbal learning and memory and the executive functions [7], among subjects with CD [24], [8], adolescent girls [25–26] and those with comorbid bipolar disorder [27]. However, few studies have addressed problems associated with heterogeneity. Childhood- and adolescent-onset CD is often combined into a single group, and other disorders such ADHD may influence findings, factors taken into consideration in the present study.

### Life-Course Persistent Offenders versus Adolescent-Onset Offenders

Moffit’s dual taxonomy model [7] proposes that different aetiologies and developmental courses define the onset of offending. According to this model, life-course persistent antisocial behavior begins in early childhood and continues throughout adulthood, while offenders with adolescent-onset antisocial behavior desist in young adulthood. However, recent studies [28–30] have questioned this theory by showing similar neurophysiological profiles in both childhood- and adolescent-onset CD. For instance, Fairchild et al. [28] found impairments in emotional processing and fear conditioning in both CD subgroups, and Roisman et al. [30] found social disadvantage from infancy for children who showed antisocial behavior primarily in adolescence, challenging the assumption that adolescent-onset CD may be normative. These authors suggest that revision of the model of the development of antisocial behavior may be necessary. The current study, therefore, sought to test the validity of Moffitt’s [31] model by examining variability in violent behavior and neuropsychiatric function.

### Risk Factors for Antisocial Youth

According to Moffit’s taxonomical model, childhood-onset antisocial youth will be more vulnerable to risk factors across multiple domains including neuropsychological dysfunction [32–33], mental-health problems, poor parenting [34], substance use disorders [11], learning difficulties and poor school attendance [35], head injuries [36], [32–33] and childhood maltreatment and trauma [37–38]. Moffit’s review [6] across 47 studies, found that antisocial youth, in general, were impaired in two specific cognitive domains: language-based verbal skills and “executive” self-control functions. These studies found strong effect sizes even when “young people who are temporarily experimenting with mild delinquent acts are lumped together with young people whose antisocial behaviors are more serious, persistent or physically aggressive” [7] [39]. However, these two groups could be further conceptualized as youth with varying risk. The greater the number of risk domains, the higher the risk of violence, as violence is the “end product of a chain of events over the course of a child’s development, where risks accumulate and reinforce each other” [40–41].

More recently, Fairchild et al. [42] reviewed the developmental taxonomic theory of antisocial behavior, reporting that both CD subtypes display emotion-processing deficits, changes in brain structure and function, as well as alteration in cortisol secretion.

Research in this field clearly indicates that neuropsychiatric and developmental risk factors are integral to the aetiology of aggression and violence. However, many investigations into antisocial youth have struggled with disorder heterogeneity. Delinquency research has not always accounted for comorbidity, with dual diagnoses of conduct disorder and ADHD resulting in the poorest outcomes and strongest predictors of adult crime [7].

The current study examines psychiatric, neuropsychological and psychosocial risk factors in non-institutionalized samples. It was hypothesized that childhood-onset CD youth would display more severe psychiatric symptoms, neuropsychological deficits, including verbal and executive deficits in particular, and higher frequency of family dysfunction and child maltreatment than their adolescent-onset peers. Furthermore we expected that the early-onset group would be characterized by more violent behavior than the adolescent-onset group.

## Method

### Participants

Forty-three young people (age range: 12–21 years, M: 15.31, SD: 2.3; gender: M: 34, F: 9) who had engaged in violent and antisocial behavior were recruited for this study through Headspace Services (n = 28)—a group specializing in the assessment and early intervention in mental health problems in young people [43–44]—and a Juvenile Justice Community Centre (n = 15). Sampling from two relevant service providers allowed a sufficient sample to be recruited, and provided a broader spectrum of people with actual offending and mental-health problems. Inclusion criteria included persons aged between 12 and 21 years and DSM-IV-TR criteria for a diagnosis of conduct disorder (CD). All young people included in the study were living in the community, either within their family homes or in non-government run group homes for young people.

### Ethics Statement

This study protocol was approved by the Human Research Ethics Committee at the University of Sydney (Ref No. 02-2009/11107). Participants were informed that participation was entirely voluntary, and if they agreed to participate, that they were able to withdraw consent at any phase of the study without prejudice. Participants 18 years and older were required to sign a “Participant Consent Form”, while parents or guardians of participants under 18 years of age were required to give written consent via the “Parental (or Guardian) Consent Form” alongside consent of the child. Participants and their legal guardians were informed of the limits of confidentiality regarding offending behavior, via the information sheet provided, as well as a script read aloud to participants prior to the commencement of the clinical interview. All participants under the Juvenile Justice System were accompanied by a caseworker, who provided additional information regarding a participant’s capacity to consent. If mental-health problems were identified during the assessment process for a participant, they were offered information regarding treatment or referred to a mental-health clinician at Headspace Services, Camperdown, Sydney, Australia.

### Procedure

Psychiatric symptoms, neuropsychological deficits and psychosocial risk factors were determined using a variety of measures, which are described below. A psychiatrist or clinical psychologist conducted clinical interviews for all potential participants, and a diagnosis of CD was given if DSM-IV-TR criteria for the disorder were met. Age of onset was defined using the DSM-IV-TR [45] criteria based on the presence of three of 15 behavioral criteria, with the presence of one characteristic behavior prior to age 10 differentiating childhood-onset from the adolescent-onset subtype. An absence of any criteria characteristic of conduct disorder prior to the age of 10 years was required to meet the criteria for adolescent-onset subtype. Identification of the childhood-onset group was determined through a series of questions asked during the clinical interview. They were: “When did you first start to get into trouble with police?”; “When were you first arrested by the police?”; date of first (if any) court appearance; “When did you first start breaking into places; stealing other people’s possessions, including breaking into cars?”

Initial attempts were made to collect parent reports on symptom onset, however many young people reported fractured family backgrounds suggesting that parent reports may be unreliable. Other difficulties encountered in collecting parent reports were the non-compliance of the parent or primary carer to complete forms. A number of young people had Juvenile Justice caseworkers who provided useful information regarding a young person’s level of overall functioning.

Young people determined to be child-onset versus adolescent-onset were separated into the two groups with the former group containing 23 subjects, and the latter containing 20 subjects. Demographic information regarding a participant’s childhood experience of maltreatment, head injury, family dysfunction and severity of violent behavior was obtained through clinical interview. Evidence for a comorbid diagnosis of ADHD was determined through clinical interview.

### Psychiatric Measures

#### Kessler Psychological Distress Scale-10

The Kessler psychological distress scale (*K-10)* [46–47] is a widely used, simple self-report measure of psychological distress consisting of 10 items and scored using a five-level response scale based on the frequency of symptoms reported for each question. It is useful in the identification of individuals who need further assessment for anxiety and depression. Scores under 20 are likely to be well. Scores between 25 and 29 are likely to have moderate mental disorder and scores 30 and over are likely to have a severe mental disorder [47].

#### Depression Anxiety Stress Scale 21

The Depression Anxiety Stress Scale 21 (DASS) is a valid and reliable measure of depression, anxiety and stress separately [48–49]. Each of the three DASS-21 scales contains seven items, divided into subscales with similar content. The depression scale assesses dysphoria, hopelessness, devaluation of life, self-deprecation, lack of interest/involvement, anhedonia and inertia. The anxiety scale assesses autonomic arousal, skeletal muscle effects, situational anxiety, and subjective experience of anxious affect. The stress scale is sensitive to levels of chronic non-specific arousal. It assesses difficulty relaxing, nervous arousal, and being easily upset/agitated, irritable/over-reactive and impatient. Scores for depression, anxiety and stress are calculated by summing the scores for the relevant items. The DASS-21 was administered to determine mild = 0-4/moderate = 5-9/severe = 6–10/extremely severe scores = 11+ for each DASS scale.

#### Hamilton Depression Rating Scale

Hamilton Depression Rating Scale (HAM-D) is a clinician-administered rating scale to assess symptom severity in depressive disorders. Symptoms are rated on a severity scale in individuals otherwise diagnosed with depression. It is a questionnaire used to provide an indication of depression and as a guide to evaluate recovery. Although the HAM-D form lists 21 items, the scoring is based on the first 17 items. Eight items are scored on a 5-point scale, ranging from 0 = not present to 4 = severe. Nine are scored from 0–2. A score of 0–7 is considered to be normal. Scores of 16 or higher indicate full symptomatic status [41].

#### Brief Psychiatric Rating Scale

The Brief Psychiatric Rating Scale (BPRS) [50] is a 24-item scale for the identification and quantification of psychiatric symptoms. The instrument contains 24 ordered category-rating scales to assess positive and negative symptomatology in discrete symptom areas. The BPRS is a sensitive and effective measure both of psychopathology and of treatment-related symptom changes [51]. Suggested cut-off scores for the BPRS have usually related to the total score rather than sub-scales. A range of 31 to 40 relates to a “minimally ill” level of psychological distress; 41 to 53 relates to “moderately ill”; and above 53 is considered “markedly ill” level of psychological distress [52–53]. The BPRS Total score will therefore be used to determine cut-offs for the two groups.

### Neuropsychological Measures

Trained research psychologists administered a battery of neuropsychological tests covering a number of cognitive domains including Intellectual Ability, General Knowledge, Processing Speed, Simple Attention, Sustained Attention, Working Memory, Learning & Memory—Verbal, Learning & Memory—Visual, Visual Spatial, Executive Functioning, Cognitive Flexibility, Executive Functioning—Verbal Fluency. Measures were combined into composites if they measured similar areas of cognitive functioning. Test scores were converted to z-scores to ensure common means and standard deviations and then summed and averaged. All the tests had recent norms that are representative of the age and educational status for the population under investigation. All the instruments were well standardized, reliable and validated in prior studies. The test battery was designed to assess: intellectual ability, speed of information processing, working memory, executive function, planning and organization, simple and sustained attention, visual spatial skills, visual and verbal learning and memory and processing speed. Table 1 is a description of how neuropsychological measures were interpreted using standardized scores [46].

**Table 1:** Description of standardized scores for neuropsychological tests.

Description	Z Scores
Very superior	≥2.00
Superior	1.30 to 1.99
High Average	0.68 to 1.29
Average	-0.68 to 0.67
Low Average (Mildly Impaired)	-1.29 to -0.67
Borderline (Moderately Impaired	-1.99 to -1.30
Extremely Low (Severely Impaired	≤-2.00

#### Wechsler Test of Adult Reading

The Wechsler Test of Adult Reading *(WTAR)* [54] consists of a word reading list and estimates IQ. It has been co-normed with the third editions of the Wechsler Adult Intelligence and Memory Scales. The WTAR also has the advantage of offering three methods by which to estimate IQ, based on reading performance, demographic information or a combination of the two. In the design of the WTAR, the demographic prediction tables were co-normed with the widely used Wechsler Adult Intelligence Scale (WAIS) and Wechsler Memory Scale (WMS). The *Wider Range Achievement Test R (WRAT-R)* [55–56] is the child version of academic achievement, administered to participants 16 years and younger.

#### Wechsler Adult Intelligence Scale-III

The Wechsler Adult Intelligence Scale-III (WAIS-III) *Information i*s a subscale of the verbal IQ score and is a measure of general knowledge [55]. The *Wechsler Intelligence Scale for Children-III (WISC-III) Information* was administered to participants 16 years and younger.

#### Trail-Making Test

The Trail-Making Test (TMT) is a measure of attention, speed and mental flexibility. It consists of parts A and B. Both parts of the Trail Making Test consist of 25 circles distributed over a sheet of paper. In Part A, the circles are numbered 1–25, and the patient is required to draw lines to connect the numbers in ascending order. In Part B, the circles include both numbers (1–13) and letters (A–L); as in Part A, the participant draws lines to connect the circles in an ascending pattern, but with the added task of alternating between the numbers and letters (i.e., 1–A–2–B–3–C, etc.). The participant should be instructed to connect the circles as quickly as possible, without lifting the pen or pencil from the paper. Part B of the TMT has been found to be the most sensitive to frontal damage and involves the ability to alternate between, and maintain, two sets of stimuli [57].

#### Controlled Oral Word Association Test

Controlled Oral Word Association Test, abbreviated COWA or COWAT, is a verbal fluency test that measures spontaneous production of words belonging to the same category or beginning with some designated letter. The participant is asked to name words beginning with a letter, excluding proper nouns, for one minute and this procedure is repeated three times. The most common letters used are F, A, and S because of their frequency in the English language. The examiner must quickly write down the words provided by the participant on a piece of paper. Word generation has been found to be a reliable test of left frontal and executive functions [54]. The Controlled Oral Word Association Test [58–59] evaluates the spontaneous production of words under restricted conditions.

#### Cambridge Neuropsychological Test Automated Battery

The Cambridge Neuropsychological Test Automated Battery (CANTAB) is a computer-administered, nonverbal (visually presented) set of tasks developed to examine specific components of cognition. The software comprises one screening test and 12 principal tests from the CANTAB system [60–62]. The CANTAB is designed to test different aspects of mental functioning so that a profile of performance can be constructed, including independence of executive measures and memory factors [63]. The CANTAB subtests consist of: spatial span, choice reaction time, rapid visual processing, intra/extra-dimensional shift, paired associated learning. The test scores are computer generated and give a rating from impaired to high average for: simple and sustained attention, visual and verbal learning and memory, working memory, speed of information processing, visual spatial skills and executive function.

#### Verbal memory

Immediate and delayed verbal memory was measured using the Logical Memory subscale of the Wechsler Memory Scale (WMS-III) [64]. Participants were required to recall stories A and B after a 30-minute delay. The examiner records the number of free recall and thematic units.

#### Rey Auditory Verbal Learning Test

The Rey Auditory Verbal Learning Test (RAVLT) is a test of memory where the examiner reads a list of 15 concrete nouns. The examinee recalls as many as possible in any order through five administrations and a recognition trial. It allows for the identification of memory impairment and is a measure of verbal memory.

#### Rey Complex Figure Test

The Rey Complex Figure Test (RCFT) is a test for the evaluation of visuospatial constructional ability and visual memory. It is a tool for measuring executive function underpinned by prefrontal lobe functioning. The RCFT consists of three test conditions: Copy, Immediate Recall and Delayed Recall. Subjects are given the RCFT stimulus card and asked to draw the same figure, then asked to draw what they remember. Then after a 30-minute delay they are requested to draw the same figure again. RCFT recall is sensitive to mild neuropsychological impairment in a variety of clinical populations [58]. Traumatically brain-injured patients tend to have difficulty performing in CFT recall trials.

### Psychosocial Measures

#### Social and Occupational Functioning Assessment Scale

Psychosocial factors were measured on the Social and Occupational Functioning Assessment Scale (SOFAS) and various items on the semi-structured interview. The *SOFAS* is a clinician-administered measure of problems in social, occupational and interpersonal functioning. It measures the frequency of social activities across seven subscales: *withdrawal/social engagement*, *interpersonal communication*, *independence-performance*, *independence-competence*, *recreation*, *prosocial and employment/occupation*. It focuses exclusively on the individual’s level of social and occupational functioning and is not directly influenced by the overall severity of the individual’s psychological symptoms [46].

#### Severity of Dependence Scale

Participants were also administered the Severity of Dependence Scale (SDS) [65], which is a short, clinician-administered rating scale used to measure the degree of dependence experienced by users of different types of drugs. The SDS contains five items, all of which are explicitly concerned with psychological components of dependence. These items are specifically concerned with impaired control over drug taking and with preoccupation and anxieties about drug use. Higher scores indicate higher levels of dependence. It is primarily a measure of compulsive use, which is a central component of dependence.

#### Family Dysfunction Measures

Three household dysfunction variables were used in the study: Household mental illness, Household substance use disorders and Household learning disability, all binary, self-report measures. Each variable is comprised of information taken from the subject’s family history. Many participants tended to be poor historians, therefore it was difficult to quantify the number of family members affected and the severity of the disorder for each family member on each variable.

*Household mental illness* refers to the degree of mental-health problems in the young person’s immediate family. Mental-health problems include mood and psychotic disorders. *Household SUDS* refers to the incidence of drug and alcohol use in the young person’s immediate family. *Household learning disability* refers to the incidence of learning disabilities, including autism spectrum disorders and Asperger’s disorder in the young person’s immediate family. There were a number of self-report measures taken during the clinical interview that were included in the analysis as binary social/environmental variables. These include: *Childhood physical abuse*, *Incidence of head injury*, *Substance use and School attendance*.

#### Severity of conduct disorder

A binary variable measuring the degree of aggression and violence the young person has engaged in. This measure relates to the “Severity Specifiers” for conduct disorder categorization in the DSMIV-TR.

*Level 1* is a mild to moderate level of violence and antisocial behavior and includes damage to property, initiating physical fights either in the home and school, bullying and threatening behavior; truanting from school, school suspensions and expulsions, aggression toward others.

*Level 2* is a more severe level of violence and antisocial behavior and includes serious assaults leading to charges and convictions. Offences include break and enter, use of a weapon, armed robbery and attempted murder.

### Data and Statistical Analysis

All statistical analyses were performed using SPSS, version 20 (SPSS Inc., Chicago, Illinois, USA). The various psychiatric, neuropsychological and psychosocial variables were subjected to independent t-test (continuous variables) and chi square (for categorical variables) analyses to determine whether early- and late-onset antisocial youth could be distinguished on specific risk factors identified in the literature. Participants were excluded from the study at the point of statistical analysis if they were identified as an outlier deemed to be 1.5 times the interquartile range on all neuropsychological measures. All language-based neuropsychological tests were corrected for years of education within standardized scoring calculations and were appropriately normed. Significant effects were set at *p* < .005 for *t* tests and chi-square allowing for a delicate balance between Type I and Type II errors. Cohen’s d effect size statistics were calculated for each pair-wise comparison consistent with efforts towards “meta-analytic thinking” [66]. Cohen’s guidelines [67–68] identify 0.2, 0.5, and 0.8 as small, medium, and large effects, respectively. Odds ratios were calculated for chi square statistics indicating the degree of association between binary variables.

### Neuropsychological Composites

#### Composite measure of executive function

A composite measure of variables was created using the SPSS “compute variable” procedure to measure the construct “executive function” across the delinquency group. Three neuropsychological tests, namely Trail Making Test A and B, Intra/Extra Dimensional shift and COWAT animals and letters were included in the composite as they each measure various aspects of executive function [58]. The Trail Making Test A and B measures visuo-motor tracking, divided attention and cognitive flexibility and is sensitive to frontal lobe lesions [57]. Word fluency and the generation of word lists on the Controlled Oral Word Association Test, F-A-S, is a sensitive indication of brain dysfunction, particularly within the frontal area. People with frontal-lobe lesions have reduced letter and category fluency and therefore deficient retrieval strategies. Intra-extra Dimensional Shift is a test of rule acquisition and reversal. It measures the visual discrimination, attentional set formation maintenance, shifting and flexibility of attention and, therefore, is primarily sensitive to changes in the frontal areas of the brain [69].

#### Composite measure of auditory verbal learning and memory (RAVLT)

Comprised items on the Rey Auditory Verbal Learning and Memory test: RAVLT sum, A6 and A7 and produced through the SPSS “compute variable” procedure. A6–A7 measures susceptibility to proactive and retroactive interferences and correlates moderately with measures of immediate recall (Sum A1-A5) [58].

### Psychiatric Composite

The BPRS Total score was used as a general measure of psychiatric symptoms.

### Psychosocial Composite

#### Composite measure for family dysfunction

Comprised the three Household Dysfunction measures: Household mental illness, Household SUDS and Household learning disability, which were manually collated from the categorical dataset.

## Results

### Participant Characteristics

Among the 43 young persons assessed (age range: 12–21 years, M: 15.31, SD: 2.3; gender: M: 34, F: 9), no significant differences between age-of-onset groupings were found regarding the subject’s age at assessment (t(41) = -1.02, p = .31), or for diagnosis of ADHD χ*2*(2, N = 43) = 4.6, *p* = .10). Of the 23 early-onset youths, 16 were diagnosed with comorbid ADHD. The late-onset group, comprising 20 participants, had 13 individual with comorbid ADHD. There were no significant differences observed between groups for gender, (χ*2*(1, N = 43) = 1.8, *p* = .17), with the early-onset group containing three females versus six females in the late-onset group. Among the youths, those with early-onset CD had significantly lower levels of education than did the late-onset youths (t(41) = -2.35, *p =* .02). Table 2 refers to means, standard deviations, effect sizes and frequencies for participant characteristics with odds ratios and confidence intervals at 95% for nominal data.

**Table 2:** Participant Characteristics.

	Childhood-onset CD	Adolescent-onset CD	Cohen’s d
	(M ± SD)	(M ± SD)	
*N*	23	20	
**Gender**	M: 20 (87%)F: 3 (13%)	M: 14 (70%)F: 6 (30%)	
**Age at Assessment**	15 ± 2.29	15.75 ± 2.51	.3
**Years of Education**	8.39 ± 1.67	10 ± 2.75	.71
**Subjects Age at Assessment**			
**Years**			**Frequency**
12	4	1	5
13	2	2	4
14	4	4	8
15	5	5	10
16	2	1	3
17	3	3	6
18	2	1	3
19		1	1
21	1	2	3
**Total**	**23**	**20**	**43**

### Psychiatric data

Groups differed significantly on the BPRS total (t(38) = 2.5, *p =* .01) with the early-onset group exhibiting more psychotic like symptoms, such as hallucinations, delusions, disorientation, mania and negative symptoms. Both groups fell within the “minimally ill” level, however the early-onset group were further along the scale toward the “moderately ill” level. The groups also differed significantly on the YMRS (t(23) = .2.6, p = .001, with both groups falling below the ≤12 cut-off score of threshold symptomatology. No significant differences were observed between the groups on the DASS21, with depression (*t*(36) = .13, *p* = .81, anxiety (*t*(37) = .67, *p* = .83 and stress (*t*(37) = .78. *p =* .76, falling within the moderate to severe range in symptom severity; while both groups fell within the “minimally ill” range on the Kessler-10 (*t*(38) = -.43, *p* = .28, and the HAMD (t(38) = 1.4, *p* = .4.

The groups showed significant differences with regard to “Severity of Conduct Disorder” (χ*2* (1, 43) = 7.3; *p* = .007) and “Contact with Law Enforcement Agencies” (χ*2* (1, *N* = 43) = 8.2; *p =* .006) with the early-onset group committing more serious and violent offences [Childhood onset: 14 (77.8%); Adolescent onset: 4 (22.2%)], as well as having more contact with police and the juvenile court system [Childhood onset: 18 (78%)]; Adolescent onset: 8 (40%)]. There were no significant differences for the group regarding “Severity of Dependence-Primary Drug” (χ*2* (3, 43) = 5.0; *p =* .17), and “Head Injury” (χ*2* (3, *N* = 43) = 2.1; *p =* .15),

### Neuropsychological data

Groups differed significantly on the RAVLT composite (t(41) = -3.3, *p =* .002) with the early-onset group performing worse on these measures. No significant differences were observed between groups on WTAR/WRAT (t(41) = -2, *p =* .05), WAIS/WISC Information (t(40) = -1.5, *p =* .15), Choice Reaction Time—simple movement time (t(34) = 1.58, *p =* .12),—simple reaction time (t(36) = -0.21, *p =* .05),– 5 choice movement (t(36) = -0.21, *p =* .83),– 5 choice reaction (t(36) = -1.24, *p =* .22), Mental Control/Sequences (t(40) = -0.16, *p =* .87), Rapid Visual Processing A (t(38) = -1.6, *p =* .12), Rapid Visual Processing B (t(35) = -2.0, *p =* .05), Rapid Visual Processing mean latency (t(36) = -1.7, *p =* .09), Paired Associate Learning-total errors adjusted (t(39) = -0.94, *p =* .35), Paired Associate Learning-total errors 6 shapes (t(38) = -0.53, *p =* .59), spatial span length (t(39) = -2.6, *p =* .01), Trail Making Test A (t(39) = -1.73, *p =* .04 and B (t(41) = -1.5, *p =* .3, Logical Memory 1 (t(17) = -1.2, *p* = .04, 2 (t(17) = -1.9, *p =* .04 and Rey Auditory Verbal Learning B1 (t(41) = -1.04, *p =* .4 and Executive Function composite (t(36) = -1.38, *p =* .17).

Score interpretation (Table 1) shows that the RAVLT (M = -3.13 and SD = 3.23) for early-onset CD fell within the Extremely Low (Severely Impaired) range while the late-onset CD fell within the Average Range score (M = 0.18 and SD = 3.35). Both the early-onset (M = -5.6 and SD = 7.5) and late-onset (M = -2.2 and SD = 7.3) scores for “Executive Function” fell within the Extremely Low range (Severely Impaired) with no significant difference between the two groups. Mean scores for the remaining neuropsychological tests, including IQ, fell within the average to below-average range of functioning.

### Psychosocial data

Significant differences were observed between groups for “Childhood Physical Abuse” (χ*2* (1, *N =* 37) = 9.9; *p <*.005) with child abuse being more frequently observed in conjunction with childhood-onset CD [Childhood-onset: 12 (67%); Adolescent-onset: 3 (16%)]. There were no significant differences for “Current Living Arrangements—living in a single parent household” (χ*2* (3, *N =* 42) = 7.1; *p =* .06), “Household Mental Illness” (χ*2* (2, *N =* 43) = 1.2; *p =* .55), “Household Learning Disability” (χ*2* (2, *N = 43) =* 2.1; *p =* .35) and “Household SUDS” ((χ*2* (2, *N* = 43) = 1.6; *p =* .45). Table 3 refers to means, standard deviations and effect sizes for neuropsychological and psychiatric measures and psychosocial risk factors with odds ratios and confidence intervals at 95% for nominal data.

**Table 3:** Neuropsychological, Psychiatric and Psychosocial Measures.

	CO CD (M ± SD)	AO CD (M ± SD)	Cohen’s d
	Childhood onset	Adolescent onset	
**PSYCHIATRIC MEASURES**			
BPRS total	38.7 ± 9.87	31.7 ± 6.64	.83
ADHD	16 (70%)	13 (65%)	
YMRS	6.36 ± 9.2	0.7 ± 1.9	.85
HAMD	7.0 ± 5.3	4.8± 4.9	.43
Kessler-10	19.8 ± 5.6	20.8 ± 8.3	.14
DASS depression	9.6 ± 11.1	9.1 ± 9.9	.04
DASS anxiety	7.1 ± 6.4	5.7 ± 6.4	.22
DASS stress	13.5 ± 10.4	10.8 ± 11.3	.25
Severity of CD	14 (77.8%)	4 (22.2%)	
Severity of Primary Drug Dependence	12 (66.7%)	6 (33.3%)	
**NEURO- PSYCHOLOGICAL DATA**			
Head Injury	9 (69.2%)	4 (30.8%)	
RAVLT composite	-3.13 ± 3.23	0.18 ± 3.35	1.00
Spatial Span	-0.68 ± .81	0.10 ± 1.10	.81
Executive function composite	-5.6 ± 7.5	-2.2 ± 7.3	0.5
**PSYCHOSOCIAL DATA**			
Childhood Abuse	12 (67%)	3 (16%)	
Contact with law enforcement	18 (78%)	8 (40%)	
Household dysfunction composite	4.6 ± 0.93	5.1 ± 1.2	0.5

BPRS: Brief Psychiatric Rating Scale; ADHD: Attention Deficit Hyperactivity Disorder; YMRS: Young Mania Rating Scale; K-10: Kessler-10; DASS: Depression Anxiety Stress Scale; RAVLT: Auditory Verbal Learning and Memory composite.

## Discussion

The current study examined psychiatric, neuropsychological and psychosocial risk factors in distinguishing childhood- from adolescent-onset CD. Childhood-onset conduct disorder was characterized by: (1) impairment of verbal learning and memory (as indicated by the RAVLT); (2) higher reporting of childhood physical abuse; (3) higher rates of mental-health problems, specifically psychotic-like symptoms, but not depression and anxiety; (4) lower levels of education; (5) more contact with police and juvenile justice agencies; and (6) committing more serious, violent offences. All findings were associated with large effect sizes.

Childhood-onset CD youths displayed global cognitive impairment across executive function, verbal learning and memory. They were significantly more likely to suffer from neuropsychological deficits measured by the RAVLT, a finding associated with a large effect size. The RAVLT evaluates verbal learning, memory and auditory processing, and includes functions such as proactive inhibition, retroactive inhibition, retention, encoding versus retrieval and subjective organization [54]. Performance IQ was observed to be greater than Verbal IQ in a number of delinquency studies which suggests childhood onset CD youth may suffer from a specific deficit in language manipulation. Verbal deficits affect receptive listening and reading, problem solving, expressive speech, writing and memory for verbal material [70] and it has been suggested that verbal ability is a necessary skill for self-control of behavior, as it influences the success of socialization, beginning with parent-child interactions [7–8]. Our results support previous findings as subjects performed poorly in language-based neuropsychological tests and memory tests, but not in non-language-based tests. Our study provides an important contribution as it focused on a more homogeneous CD group, rather than relying on a delinquency cohort.

Both childhood- and adolescent-onset groups were in the “severely impaired” range for executive functioning. Both verbal and executive-function deficits are likely to contribute to the antisocial behavior in these groups, reducing the child’s ability to control their own behavior and therefore act out impulsively. These results suggest a shared vulnerability, with dimensional differences in brain development related to executive function between the two groups. Any degree of impairment with cognitive function is likely to place a young person at risk of impulsive behavior and poor decision-making. Our results suggest that adolescent-onset CD may not be a normative process, as the adolescent-onset sub-group also displays impairment in executive function. This is consistent with previous studies [28–30] showing similar neurophysiological profiles in both childhood- and adolescent-onset CD.

Both childhood- and adolescent-onset groups reported moderate to severe depression, anxiety and stress symptoms using a range of depression and anxiety measures, with no significant differences between the two groups. Childhood-onset youth had a significantly higher mean score on the Young Mania Rating Scale and the Brief Psychiatric Rating Scale than the adolescent-onset youth, although they fell below the symptom threshold for both measures. Three subjects (two childhood-onset and one adolescent-onset) who reported psychotic symptoms at the time of interview, had previously used cannabis or hallucinogens and it was suspected that in these cases, psychotic symptoms were substance induced, with symptoms remaining following the cessation of substance use. Psychiatric symptoms, particularly first-episode psychosis, have been linked with violent behavior in a number of studies [11–13], [71–72]. Although mean scores were sub-clinical, this study provides some support for fluctuating mood and psychosis emerging in early-onset youth.

Psychosocial risk factors can significantly impact and increase the risk of developing chronic conduct problems. Parental antisocial personality disorder, alcohol dependence, mood disorders and schizophrenia have been found to be higher for childhood-onset CD. Findings from our study did not indicate significant differences between groups for family risk factors, although childhood-onset youths were more likely to have experienced childhood physical abuse than their adolescent-onset peers, a finding associated with a large effect size. Childhood-onset youths also reported significantly fewer years at school. It was anticipated that early-onset youths would have higher rates of substance use than adolescent-onset youths, however, the Severity of Dependence Primary Drug scale did not demonstrate differences between the two groups. This could be due to legal issues related to reporting or abstaining from substance use whilst a young person is under a community treatment order or parole conditions. Childhood-onset youths were also more likely to have contact with police and juvenile justice agencies as well as committing more serious, violent crimes. Overall, there was a relationship between auditory verbal learning and memory, child abuse and childhood-onset CD independent of other risk factors.

The strengths of the study include a sample population of CD youth distinguishing for age of onset based on the presence of behavioral difficulties. This reduces the heterogeneity usually associated with measuring risk factors in antisocial youth populations, a significant strength of our study. Previous studies have focused on delinquent populations in custody rather than community settings. Few studies have examined subjects with a diagnosis of conduct disorder. There are important distinctions between the two groups, as conduct disorder refers to a mental disorder and juvenile delinquency to a legal status. Juvenile delinquency is more prevalent than conduct disorder. A designation of juvenile delinquency only requires participation in one illegal act [73]. Time of onset for delinquency groups is, therefore, arbitrarily based on criminal charges rather than the onset of antisocial behavior. Focusing on the diagnosis of conduct disorder however—as we do here—provides a more homogeneous group for study. Furthermore, delinquency research has not always accounted for comorbidity, with dual diagnoses of conduct disorder and ADHD resulting in the poorest outcomes and strongest predictors of adult crime [7]. In the current study, there were no significant differences for ADHD between the two groups, suggesting that observed differences were related to age of onset of CD, rather than the presence/absence of ADHD.

The limitations of the study include a relatively small sample size, measures of substance use that do not reflect usage at the time an offence was committed and demographic measures based on self-report. Collaborative information regarding a subject’s family history would allow for better discrimination of these factors in future studies. The two sample groups were also overwhelmingly male, an observation that is representative of the CD population [74]. Additionally, information on ethnicity and socioeconomic status was not consistently recorded for participants. Finally, it is noted that testing occurred prior to the publication of DSM-5, therefore we did not assess participants on capacity for prosocial emotions, which is now a specifier for conduct disorder diagnosis in DSM-5. This specifier may help to identify youths characterized by callous-unemotional traits.

In conclusion, our study reveals that childhood- and adolescent-onset CD differed for a number of psychiatric, neuropsychological and demographic risk factors. Childhood-onset CD performed more poorly than adolescent-onset CD for auditory verbal learning and memory tasks, but did not differ for measures of executive function. Both groups exhibited severe impairment on executive function tasks challenging theory indicating that adolescent-onset CD may be a normative process. Childhood-onset CD also exhibited more psychotic-like symptoms than adolescent-onset CD. Those with childhood-onset CD reported child abuse more frequently. Childhood-onset youths had more frequent contact with juvenile justice agencies and they committed more serious acts of violence. This study is unique as it integrates risk factors across psychiatric, neuropsychological and psychosocial domains of function in a CD population, distinguishing for both the time of onset of CD as well as comorbid ADHD. Further investigation into CD subtypes, such as CD and comorbid ADHD and CD alone, are necessary to distinguish unique risk factors amongst sub-groups. Children with ADHD are more likely to receive a comorbid diagnosis of oppositional defiant disorder and conduct disorder and they are more likely to have written language disorders and executive function deficits [75]. The two groups in the present study did not differ in rates of ADHD and were therefore controlled for, however the small number of subjects in the subgroups limited the power of the analysis.

In conclusion, our findings provide partial support for Moffit’s dual taxonomy model in that childhood-onset youth were found to exhibit vulnerabilities across multiple risk factors. Children with deficits in verbal skills and executive function who are experiencing physical trauma and childhood abuse are more likely to experience behavioral problems that set the stage for developing violent and antisocial behavior. However, our findings also challenge Moffit’s “normative” theory of adolescent onset antisocial behavior, providing support for Fairchild’s [28] developmental theory. Our findings highlight the need for further investigation in larger samples.
